# Altered PAPP-A and Placental Thickness in Pre-Eclampsia and Intrauterine Growth Restriction: A Pilot Study

**DOI:** 10.3390/jcm15041607

**Published:** 2026-02-19

**Authors:** Liviu Moraru, Raluca Moraru, Melinda Ildiko Mitranovici, Romeo Micu

**Affiliations:** 1 Department of Anatomy, George Emil Palade University of Medicine, Pharmacy, Sciences and Technology, 540142 Targu Mures, Romania; liviu.moraru@umfst.ro (L.M.); raluca.moraru@umfst.ro (R.M.); 2 Doctoral School of Medicine and Pharmacy, George Emil Palade University of Medicine, Pharmacy, Science, and Technology of Targu Mures, 540142 Targu Mures, Romania; 3 Department of Obstetrics and Gynecology, University of Medicine and Pharmacy Iuliu Hatieganu, 400347 Cluj-Napoca, Romania; romeomicu@hotmail.com

**Keywords:** pre-eclampsia, intrauterine growth restriction, PAPP-A, placental thickness

## Abstract

Pre-eclampsia (PE) is a multisystem disorder that affects 5–6% of all pregnancies. **Background**: PE and intrauterine growth restriction (IUGR) are two major causes of maternal mortality and morbidity. We hypothesize that first-trimester pregnancy-associated plasma protein-A (PAPP-A) levels are useful as a prognostic marker. The aim of our study is to identify the role of PAPP-A and placental thickness in pre-eclampsia screening, as well as its value in IUGR prognosis. **Methods**: This prospective study was conducted at the Al. Simionescu County Hospital Hunedoara, Romania, Department of Obstetrics and Gynecology, from 12 May to 31 October 2025. A total of 102 patients were included in our study; of these, 28 patients (28.56%) developed pre-eclampsia, and 13 (13.26%) developed IUGR associated with PE. **Results**: The demographic data showed no differences between groups, except for BMI, smoking habits, and diabetes mellitus. Of the 28 cases of pre-eclampsia, 14.28% had PE detected by 28 weeks, 46.4% had PE associated with IUGR by 33/34 weeks, and 39.32% had PE detected at term/delivery. The highest detection rate was at 33/34 weeks, when the association with IUGR was obvious. For PE with IUGR at 34 weeks, the area under the curve (AUC) was 0.909, with a *p*-value < 0.001. The PAPP-A cut-off was 0.65 MoM, indicating high sensitivity and specificity for predicting PE. Placental thickness was also assessed, resulting in statistically significant differences between groups. The PAPP-A level shows strong predictive value for PE, especially when associated with placental thickness. **Conclusions**: This study demonstrates a clear correlation between low PAPP-A in the first trimester of pregnancy, placental thickness in the second trimester, and the subsequent development of pre-eclampsia and its association with IUGR.

## 1. Introduction

Pre-eclampsia (PE) is a multisystem disorder that occurs after 20 weeks of pregnancy, affecting 5–6% of all pregnancies [1,2]. It is diagnosed according to an elevated blood pressure > 140/90 mmHg with proteinuria higher than 300 mg in a 24 h urine sample [1,2,3]. PE and intrauterine growth restriction (IUGR) are among the major causes of perinatal mortality and morbidity [4]. Biomarkers to predict pre-eclampsia would be extremely useful to ensure early diagnosis [2], and a range of angiogenic factors have been examined for this purpose [5]. It is hypothesized that PAPP-A serum levels in the first trimester of pregnancy are correlated with soluble fms-like tyrosine kinase-1 (sFlt-1) levels and can be useful as prognostic markers [6].

Several researchers have demonstrated that the risk of developing pre-eclampsia can be evaluated by using biomarkers such as the placental growth factor (PlGF), pregnancy-associated plasma protein-A (PAPP-A), maternal characteristics, and the first-trimester uterine artery pulsatility index [7,8,9,10,11].

Pregnancy-associated plasma protein A (PAPP-A) is produced by growing trophoblasts [12]. PAPP-A is a syncytiotrophoblast-derived protease that has been linked to different disorders, such as stillbirth or fetal anomalies [13]. Although there is higher expression of PAPP-A in the placenta compared with other tissues, its potential role in placental pathogenesis in the case of PE remains unclear [14]. Several studies have demonstrated a correlation between decreased PAPP-A levels during the first trimester of pregnancy and a high occurrence of pre-eclampsia [12,15], which is why PAPP-A has been identified as a possible biomarker for pre-eclampsia [3,13]. DNA methylation [16] and persistent placental hypoxia change the expression of PAPP-A in the placenta [17,18].

Other researchers have examined placental thickness as a tool to predict pre-eclampsia. For example, Vachon Marceau observed a significantly increased placental thickness between 11 and 14 weeks of pregnancy in patients who developed pre-eclampsia [19], while Kumar observed the opposite [20].

The aim of our study is to identify the role that PAPP-A plays in pre-eclampsia screening, the role of placental thickness, and their value in IUGR prognosis.

## 2. Materials and Methods

This prospective study was conducted at the Al. Simionescu County Hospital Hunedoara, Romania, Department of Obstetrics and Gynecology, from 12 May to 31 October 2025. In this study, pre-eclampsia was determined as blood pressure > 140/90 mmHg associated with proteinuria ≥ 300 mg in a 24 h urine sample [1,2,3,4]. IUGR was defined as an estimated fetal weight below the 10th percentile for gestational age and gender, in combination with Doppler velocimetry of the uterine and umbilical artery [12,21]. We included pregnant women with singleton pregnancies, recruited in the second trimester, before they reached 28 weeks of gestation. All patients had their PAPP-A levels measured in the first trimester of their pregnancy; this measurement was performed using a blood sample from the mother. Pregnant women with unclear gestational age, a loss of follow-up, infections, premature rupture of membranes, non-hypertensive abruptio placentae, preterm labor, alcohol/drug use, genetic abnormalities or congenital anomalies, or multiple pregnancies were excluded. Written informed consent was obtained from all women enrolled in this study. Patients who did not sign the informed consent were excluded. The inclusion criteria for the control group were pregnant women who agreed to participate in the study regardless of their risk factors for PE, with PAPP-A measured in the first trimester of their pregnancy. These selection criteria allow for comparisons of the study group with pregnancies in general.

This research was approved by the Ethics Committee of George Emil Palade University of Medicine, Pharmacy, Sciences, and Technology (no. 3761/30 April 2025).

Demographic data, including key comorbidities such as pre-existing or gestational diabetes, chronic or gestational hypertension, and renal disease, were recorded.

### Timing of the Measurements

An immunoassay method (ELISA) was used to determine the PAPP-A levels in the first trimester of pregnancy. The PAPP-A values were recorded in multiples of median (MoM) from the marker report.

Placental thickness was assessed via abdominal ultrasound using 6–4 MHz probes from Siemens (Siemens Medical, Erlangen, Germany) at study entry. The cut-off value used to define a thick placenta varies with gestational age. Second-trimester placental thickness was measured between weeks 20 and 24. We set the threshold as >40 mm to serve as a PE marker, and a thickness below <1.5 cm was used to indicate placental insufficiency. The placenta was measured perpendicular to the uterine wall at the cord insertion site, in the sagittal view from the fetal side of the chorionic plate to the maternal side of the placental–myometrial interface in the area of cord insertion [22].

**Figure 1:**
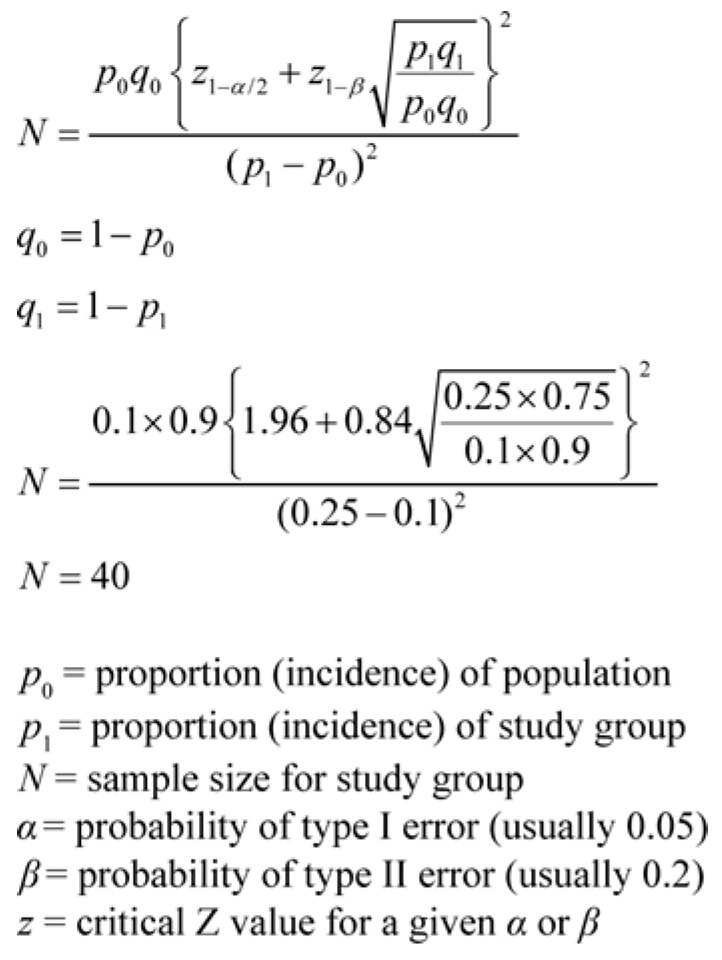
Sample size calculation.

Doppler velocimetry was performed from the umbilical artery and uterine arteries in the third trimester of pregnancy at 28 and 33/34 weeks. After a minimum of five consecutive waveforms, the abnormal end-diastolic blood flow velocity was recorded. This is a prospective observational study; we define the primary objective as follows: the presence or absence of disease (dichotomous parameter). A standard formula was used:

The sample size required was 40, according to our study design (Figure 1). We included 102 patients, thus enabling quantitative research based on this sample size. The research presented is descriptive and relational, providing data that allow for statistical analysis.

Statistical significance was set at *p* < 0.05. Statistical analysis was performed using the Statistical Package for Social Sciences (SPSS, version 23, Chicago, IL, USA). Data were labeled as nominal or quantitative variables. Nominal variables were characterized by using the means of frequencies. Quantitative variables were tested using the Kolmogorov–Smirnov test and were described by the mean ± standard deviation. The frequencies of nominal variables were compared using a chi-square test. Differences in the mean between groups were analyzed using a *t*-test. Serum PAPP-A levels were compared in each group and between groups using Student’s chi-square *t*-test and ANOVA tests. The cut-off points were defined using ROC (receiver operating characteristic) curves, which were generated for the PAPP-A MoM data.

## 3. Results

A total of 102 patients were included in our study; of these, 28 patients (28.56%) developed pre-eclampsia, and 13 (13.26%) developed IUGR associated with PE. The control group contained patients with normal pregnancies who met the inclusion criteria.

### 3.1. Demographic Data

As presented in Table 1, demographic data were recorded. For the quantitative variables (age, BMI) between the three groups, the ANOVA test and the multiple comparison Bonferroni test were applied, showing statistically significant differences between the groups regarding BMI (*p* = 0.01). BMI was statistically significantly higher in the IUGR and PE groups versus the control. For qualitative variables, the chi-squared test was applied with statistical significance for smoking habits (*p* = 0.009) and chronic diseases such as diabetes mellitus (*p* = 0.001). This indicates that the group containing patients with PE and PE + IUGR has a higher incidence of smoking and diabetes mellitus.

### 3.2. Incidence and Timing of PE/IUGR

**Figure 2:**
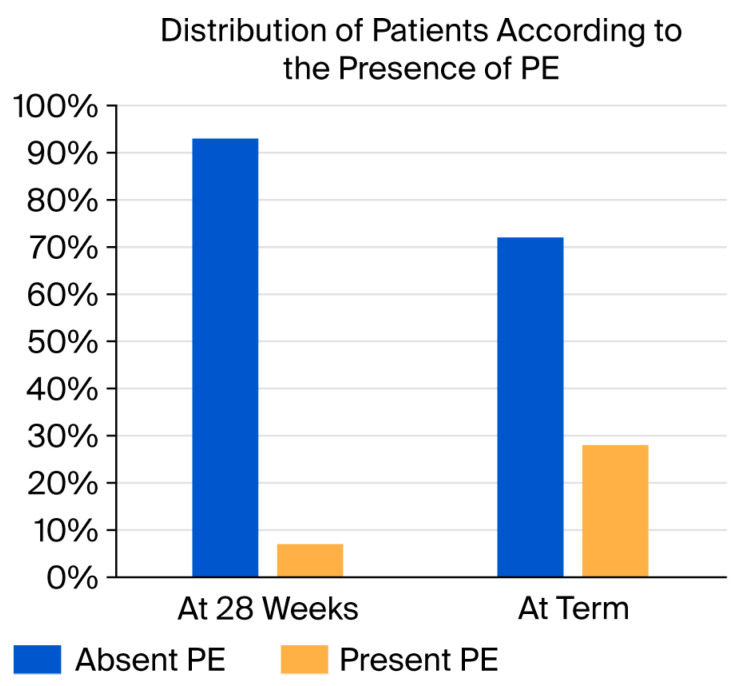
Distribution of patients according to presence of PE (3.92% at 28 weeks; 28.56% at term).

Out of the 102 patients recruited, by 28 weeks of gestation, 4 patients (3.92%) developed PE; by 33/34 weeks, 13 patients developed IUGR associated with PE (13.26%); and 11 patients developed PE only at term/delivery. A total of 28 patients (28.56%) had pre-eclampsia, with the risk increasing as the pregnancy progressed. We did not encounter severe PE. This emphasizes the crucial role of monitoring women for pre-eclampsia during pregnancy; in many cases, the diagnosis is made when pre-eclampsia has already occurred (Figure 2).

Out of the 28 cases of pre-eclampsia, 14.28% were detected at 28 weeks, 46.4% were associated with IUGR at 34 weeks, and 39.32% had PE detected at term (Table 2).

### 3.3. Doppler and Placental Findings

These data show that most of the PE cases in our study were identified in the third trimester. The Doppler velocimetry data recorded at 33 weeks of gestation identified differences in umbilical artery pulsatility indices (PIs), which were significantly higher in the group with IUGR associated with PE than in the pre-eclamptic group (*p* < 0.05). This observation emphasizes the severity of placental malperfusion in cases of PE with IUGR. The PI of the uterine arteries showed no statistically significant differences. Placental thickness was measured at study entry in the second trimester and showed a significant association with PE (*p* < 0.05) (Table 3).

### 3.4. PAPP-A Predictive Value

The mean PAPP-A level was 0.86 MoM, and the standard deviation (SD) was 0.45. While the mean level was within the normal range, there was significant variation in the SD between patients. Lower PAPP-A levels showed a higher probability of being associated with an IUGR risk.

The highest detection rate was at 33 weeks, when PE’s association with IUGR was obvious. The third-trimester evaluation, especially at 33/34 weeks, is of great importance for the detection of this pathology. For PE with IUGR at 33 weeks, the area under the curve (AUC) = 0.909, with a *p*-value < 0.001, and the best PAPP-A cut-off was 0.45 MoM (78.1% sensitivity and 81.2% specificity). In our research, the mean PAPP-A level was 0.862 MoM with an SD of 0.45. A PAPP-A cut-off of 0.65 MoM resulted in 83.6% sensitivity and 83.2% specificity for predicting PE, indicating that PAPP-A has strong predictive value for PE, even greater in the case of PE associated with IUGR at 33 weeks. The high AUC values indicate good discriminatory power. Statistical significance was based on the chi-square analysis (*p* = 0.001), which revealed that there was a statistically significant correlation between low PAPP-A levels and PE, especially in association with IUGR. An odds ratio of 0.041 showed a protective value of normal PAPP-A levels against PE. The association of the mean PAPP-A level with PE is presented in Table 4.

Therefore, the results suggest that PAPP-A is a good screening tool for PE, proving to be useful for risk assessment and continuous monitoring of PE during pregnancy. To emphasize this aspect, we added a continuous variable analysis using an independent samples *t*-test for the study versus control groups. This analysis identified statistically significant differences in BMI, Hba1c as a marker of diabetes mellitus, Doppler velocimetry, placental thickness, and PAPP-A levels (Table 5).

To reduce bias, multivariate analysis was carried out using linear regression. The multivariable model’s performance was described by the Hosmer—Lemeshow goodness-of-fit test. The dependent variable is PAPP-A and the independent variables are detailed in Table 6 and Table 7, as follows:

PAPP-A is statistically significantly influenced by BMI (Table 6) and placental thickness (Table 7), while the other independent variables are confounding factors.

## 4. Discussion

In this study, we investigated the correlation between PAPP-A levels in the first trimester of pregnancy, the role of placental thickness, and the development of PE alone and in association with IUGR. A significant correlation was observed between low PAPP-A levels and PE. A PAPP-A cut-off of 0.65 MoM resulted in 83.6% sensitivity and 83.2% specificity for predicting PE. Our results are in concordance with those of previous research showing that PAPP-A plays a promising role as a biomarker in PE risk assessment. In our study, we identified a mean level of PAPP-A = 0.862 MoM with an SD of 0.45, which showed important variation between patients, underlining the value of PAPP-A as a specific marker for PE risk assessment, especially in cases associated with IUGR. Our findings are consistent with those of Huang et al., who considered PAPP-A, combined with other biomarkers and Doppler velocimetry assessment, a significant predictor of PE [7].

Our study is also in concordance with Yliniemi’s study that included 64 women with early-onset pre-eclampsia who were compared with 752 women enrolled as the control group; this study demonstrated that maternal serum levels of PAPP-A in first-trimester pregnancies, together with free βHCG, AFP, PlGF, and maternal characteristics, are suitable for predicting PE [23]. In his study, Moslemi Zadeh emphasized the role played by PAPP-A and PP13 (placenta protein 13) in the first and second trimesters, with significant value for the prediction of pre-eclampsia [12]. In their study, De Villiers, Asiltas, and Jayamol came to the same conclusions [24,25,26]; however, Duan’s findings showed that serum PAPP-A, β-hCG, and alpha fetoprotein (AFP) levels were not significantly different between the PE and control groups [27]. Similarly, Atis demonstrated that the PAPP-A level had low predictive value for the severity of pre-eclampsia or HELLP syndrome [28]. In addition, according to Zhong’s review, first-trimester screening analytes, including PAPP-A, have low predictive accuracy for pre-eclampsia [29].

Bersinger et al. obtained significantly lower PAPP-A levels in women who subsequently developed pre-eclampsia [30]. Audibert et al. observed a correlation between the combination of serum biomarkers (PIGF and PAPP-A) in the first trimester, uterine artery Doppler, and the mother’s clinical characteristics [31]. According to Elshabacy, the mean pulsatility index of the umbilical artery had higher validity than PAPP-A; however, their combination proved to be a good screening method for PE [4]. Similar findings were obtained by Hoseini [32].

However, Huang, using a large cohort (340 PE pregnancies), demonstrated that the combination of PlGF, PAPP-A, and maternal features represents an accurate and cost-effective method for pre-eclampsia risk prediction [7].

Chandrasekaran observed a significant association between low PAPP-A levels and early pre-eclampsia, but not late pre-eclampsia [33]. However, according to Saruhan et al., in their research on 318 singleton pregnancies, a low PAPP-A level measured in the first trimester was not associated with IUGR or other adverse obstetrical outcomes [34]. According to Kirkegaard, the detection rate of IUGR in the presence of a low level of PAPP-A in the first trimester of pregnancy was relatively low (8–16%) [35].

We identified two different cut-offs, 0.65 MoM for overall PE and 0.45 MoM for PE with IUGR at 33/34 weeks. In two previous studies, the cut-off point for PAPP-A was very low: 0.4 and 0.35 [36]. Ethnicity or racial factors are likely to contribute to the differences in the cut-off points of PAPP-A and the incidence of IUGR [37,38]. Kirkegaard et al. determined that levels of PAPP-A observed in the first trimester of pregnancies associated with IUGR were below 0.4 or 0.5 MoM, indicating placental dysfunction. Additional ultrasound monitoring was thus recommended [35]. Masihi et al. reported that PAPP-A levels below 0.2 MoM or under the first percentile were significantly associated with adverse pregnancy outcomes, especially PE with IUGR [39].

The underlying mechanism is not very well understood; however, Christians, in his research, observed that low serum levels of placental proteins in first-trimester pregnancies that result in the development of IUGR and/or PE could be due to low exchange in the placenta and not due to reduced production [12]. In their respective studies, Gonen and Antsaklis linked low maternal serum PAPP-A with different adverse pregnancy outcomes such as preterm delivery, stillbirth, oligohydramnios, abruptio placentae, and gestational diabetes mellitus, all of which were in the context of PE and IUGR [19,40].

Tzanaki et al., in their review of 22 studies, concluded that PAPP-A can serve as a promising predictor of PE in early screening [41], and Nicolaides et al. found that low levels of PAPP-A are useful for selecting women for further Doppler investigation in pre-eclampsia risk assessment. Although PAPP-A is not included in PE screening [42], it is used in screening for fetal chromosome abnormality [43,44].

In our research, we observed an increase in PE detection rates starting at 28 weeks and through to term. We identified 14.28% of cases at 28 weeks, 46.4% cases associated with IUGR at 33 weeks, and 39.32% at term. This observation emphasizes the importance of fetal monitoring during pregnancy, especially third-trimester screening, which plays a crucial role in IUGR detection. The ROC curve analysis supports PAPP-A’s prognostic value (AUC = 0.879) for predicting PE. The optimal cut-offs for PE and IUGR (0.45 MoM) at 33 weeks compared with overall PE (0.65 MoM) differ, indicating that the timing of analysis influences the threshold, and further investigations are thus needed. It is notable that 13.26% of subjects had PE associated with IUGR. The chi-square test in our study showed a significant association (*p* = 0.001) between a low PAPP-A level and the development of PE.

In his study including 16 pregnant women with IUGR compared with 16 pregnant women without complications, Sifakis emphasized the importance of PAPP-A in predicting IUGR. However, this study was limited by the small sample size of the cohort [13]. The same observation was made by Tripathi et al. in their study including 107 women [45].

Wilson identified a link between placental gene expression and the levels of serum circulating placental-derived proteins, but the results showed that this correlation was affected by too many factors [46]. According to Camacho-Carrasco, the inclusion of novel biomarkers such as cell-free cfDNA and platelet microvesicles (MVs) adds value to the predictive performance of existing tools, which remain suboptimal [47]. Pathological changes in the placenta in PE with clinical relevance have been observed by Chiorean et al. and Moraru et al. [48,49].

Pre-eclampsia is frequently associated with abnormal placental thickness, often accompanying intrauterine growth restriction. IUGR can occur as a result of uteroplacental dysfunction, associated with PE as a predictor for clinical prognosis. According to Takahashi et al., adverse pregnancy outcomes are strongly associated with placental malperfusion [50]. While the serum level of PAPP-A in PE can indicate how well the placenta is functioning, measuring the thickness of the placenta in the first trimester can provide additional information [51]. According to Alkafrawy et al., placental thickness is associated with PE and IUGR. The authors observed that while in PE, the placental thickness is higher, in IUGR it is lower [52]. The same observation was made by Vachon-Marceau in their study who obtained values > 1.2 MoM in PE, while in IUGR, they were significantly lower, <0.89 MoM. Pregnancies complicated by both PE and IUGR were not associated with placental thickness modification compared with normal pregnancies [19]. We found a mean placental thickness in pre-eclampsia of 45.14 mm, while in the non-pre-eclampsia group, it was 32.5 mm (*p* = 0.041). Moreover, in IUGR, placental thickness was higher with a mean value of 40.12, but lower than in PE. Najafian made similar conclusions in his study, observing that second-trimester ultrasonography showed a thicker placenta in the case of PE than in the control group [53].

In Fatma Mortada Ali’s study, the authors observed the opposite: women who developed pre-eclampsia had significantly decreased placental thickness, as well as IUGR [54]. The same observation was reported in Kishwara et al.’s study, where placental thickness was low in women with PE compared with the control group [55], as well as in Kumar’s study on 44 pre-eclamptic women versus 1008 normal pregnancies [20]. Bellingeri, however, recorded lesions in placentas from pre-eclamptic women, studying the differences in placental specimens from 234 non-pre-eclamptic women and 44 pre-eclamptic women [56]. However, it was found that the cut-off value of placenta thickness varies with gestational age and the characteristics of the fetus or mother [57].

In Sun’s review, different values of placental thicknesses were presented; however, their conclusion was that PT should not be higher than 40 mm or > the 90th percentile at any stage of gestation. There is now a widely accepted cut-off for placental thickness, but clinicians acknowledge that a PT > 40 mm can be associated with adverse pregnancy outcomes, which can be linked to placental inflammation, thrombosis, fibrin deposition, and compensatory hyperplasia [57]. Karthikeyan T concluded that PT should be evaluated for any disease condition and assessed routinely during obstetric ultrasound [58].

The table below details the similarities and differences between our findings compared to the literature (Table 8).

The preferred biochemical marker for PE screening in the first trimester, according to the Fetal Medicine Foundation, is PLGF rather than PAPP-A. However, PAPP-A can be used with the same detection rate but with a higher screen-positive rate [58]. Professor Nicolaides introduced a new algorithm for the screening of PE at 11–13 weeks’ gestation [59,60]. This protocol includes maternal factors, uterine artery pulsatility index, mean arterial pressure, serum placental growth factor (PlGF), and serum pregnancy-associated plasma protein-A (PAPP-A). This prediction model achieved external validation in [60]. In our study, we added placental thickness measured in the second trimester, but the cut-off values remain to be determined.

The strength of our research lies in the finding that PAPP-A testing could be valuable in clinical practice, not only as a biomarker for pre-eclampsia but also for IUGR, potentially reducing other interventions in low-risk pregnancies. Early assessment before the establishment of placental dysfunction has high value in clinical practice, as it can helpimprove treatment and prognosis. The novelty of our research lies in the fact that it is, to our knowledge, the first study that highlights the role played by PAPP-A in combination with placental thickness in the second trimester of pregnancy in predicting pre-eclampsia and IUGR. We must look at PAPP-A as a valuable warning sign and not as a definitive diagnosis for PE. It indicates the need for close monitoring (ultrasound measurements, Doppler velocimetry, cardiotocography) of pregnant women with low PAPP-A levels. When combined with the assessment of placental thickness, its value increases significantly.

Our study is limited by its small sample size and single-center nature. Multicenter studies with a large sample size are needed to validate our findings and explore the potential of other factors. Another limitation of our study is the small number of parameters used in this predictive model. PAPP-A should be taken into consideration in combination with other parameters, including clinical assessment and ultrasound findings (Doppler velocimetry, placental thickness), to ensure a comprehensive risk assessment. Other biomarkers should be identified based on placental molecular findings.

## 5. Conclusions

This study demonstrates a high association between low PAPP-A levels in first-trimester pregnancies, placental thickness in the second trimester, and the subsequent development of pre-eclampsia. We also observed that the detection rate of PE increases progressively in association with IUGR at 33 weeks of gestation. Therefore, these findings must be integrated into the comprehensive management of at-risk pregnancies, alongside careful clinical and ultrasound monitoring.

Although PAPP-A is not routinely used on its own for screening for IUGR and pre-eclampsia, it can be used along with other markers, namely ultrasound features such as placental thickness and maternal characteristics, to determine the risk of PE. Its low levels are associated with placental dysfunction and help to identify which pregnancies require more intensive monitoring. Routine ultrasound screening in the second trimester can easily assess placental thickness and can serve as an important tool for predicting PE along with PAPP-A.

## Figures and Tables

**Table 1:** Demographic characteristics.

Demographic Characteristics	Group with PE (No. 28)	Group with PE and IUGR (No. 13)	Control Group (No. 74)	*p*-Value
Mean age (Range)	34.5 (18–41)	33.4 (18–41)	35.6 (18–39)	0.56 **
BMI	31 ± 2	33 ± 2	27 ± 3	0.01 **
Primipara	70%	82%	69%	0.74 *
Primi-gestation	67%	78%	50%	0.65 *
Education (High School)	70%	62%	62%	0.82 *
Marital Status (In a Relationship)	89%	90%	78%	0.67 *
Location (Urban)	74%	72%	50%	0.56 *
**Smoking Habit**	18%	25%	12%	0.009 *
**Diabetes mellitus**	14.3%	7.6%	2.69%	0.001
**Chronic hypertension**	3.57%	0	0	-

IUGR: intrauterine growth restriction; PE: pre-eclampsia; BMI: body mass index; * chi-squared test; ** ANOVA test.

**Table 2:** Distribution of patients according to time of PE detection and its association with IUGR.

Detection of PE	Frequency (No.)	Percent
PE at 28 weeks	4	14.28
PE with IUGR at 33 weeks	13	46.40
At term/delivery PE	11	39.32
Total	28	100

**Table 3:** Doppler velocimetry at 33/34 weeks of gestation in the groups with pre-eclampsia and pre-eclampsia with IUGR and placental thickness measurements between 20 and 24 weeks.

Artery	PE Group (n = 28)	IUGR Group (n = 13)	*p*	N (33 Weeks)
Uterine Artery PI	1.22 ± 0.71	1.66 ± 0.79	NS	0.45 (0.24–0.73)
Umbilical Artery PI	1.28 ± 0.36	1.75 ± 0.54	<0.05	1.00 (0.72–1.33)
Placental Thickness	45.14 ± 0.31	40.12 ± 0.21	<0.05	32.5 (28.6–34.7)

Data were tested for normal distribution using Student’s *t*-test. NS = not significant.

**Table 4:** Association of mean PAPP-A level with PE.

PAPP-A in MoM	PE Absent (%)	PE Present (%)	Total
<0.65	13(12.7%)	25 (23.8%)	38
>0.65	61 (59.8%)	3 (4%)	64
Total	74	28	102

**Table 5:** Bivariate analysis of combined parameters.

	Groups	N	Mean	Std. Deviation	*p*-Value
Hba1c (%)	PE	28	6.8	1.05	0.001
	Control	74	5.6	0.07	
BMI	PE	28	32.2	2.05	0.009
	Control	74	27.6	3.160	
PAPP-A in MoM	PE	28	0.62	1.223	0.018
	Control	74	0.70	1.183	
Uterine Artery PI	PE	28	1.44	0.43	0.001
	Control	74	0.45	0.28	
Umbilical Artery PI	PE	28	1.36	0.42	0.001
	Control	74	1.00	0.30	
Placental Thickness	PE	28	42.13	0.21	0.001
	Control	74	32.5	2.3	

Hba1c—glycosylated hemoglobin; BMI—body mass index; PE—pre-eclampsia; PI—pulsatility index. Student’s test: data are expressed as mean ± SD.

**Table 6:** Multivariate logistic regression, with PAPP-A as the dependent variable, and BMI, smoking habits, diabetes mellitus, and chronic hypertension as independent variables.

		B	S.E.	Wald	*p*-Value	OR	95% C.I.	
							Lower	Upper
Step 1 ^a^	BMI	0.477	0.111	18.316	0.0001	1.611	1.295	2.005
	smoking	0.116	0.678	0.029	0.864	1.123	0.297	4.241
	Hgb	0.258	1.027	0.063	0.802	1.294	0.173	9.677
	Chronic HTA	20.224	40.969	0.000	1.000	60.87	0.000	

^a^ BMI body mass index, Hgb glycosylated hemoglobin in diabetes mellitus is high, HTA high blood pressure.

**Table 7:** Multivariate logistic regression, with PAPP-A as the dependent variable, and BMI, placental thickness trim II, uterine artery PI, and umbilical artery PI as independent variables.

		B	S.E.	Wald	*p*-Value	OR	95% C.I.	
							Lower	Upper
Step 1 ^a^	Placental thick trim2	0.288	0.105	7.500	0.006	1.334	1.085	1.639
	Uter artery	0.611	0.577	1.123	0.289	1.843	0.595	5.708
	Umb artery	1.700	1.390	1.495	0.221	5.473	0.359	83.467
	BMI	−0.173	0.207	0.695	0.404	0.841	0.560	1.263

^a^ Placental thickness, Uter. Artery = uterine artery pulsatility index, Umb artery = umbilical artery pulsatility index, BMI = body mass index.

**Table 8:** Similarities and differences between our research results compared to the literature.

Articles	PAPP-A Levels in PE/IUGR [Cut-Off Value]	Placenta Thickness in PE/IUGR [Cut-Off Value]	
Our research	Low level [0.65 MoM]	Higher in PE/higher in IUGR but significantly lower compared with PE [4 cm]	
Huang et al. [7]	Low level [0.89 MoM]		
Yliniemi [23]	Low level [0.74 MoM]		
De Villiers [24]	Low level [0.70 MoM]		
Asiltas [25]	Low level [0.69 MoM]		
Jayamol [26]	Low level [14.34 µg /L]		
Duan [27]	No significant difference [0.75 MoM]		
Atis [28]	Low predictive value [0.69 MoM]		
Zhong [29]	Low predictive value [meta-analysis]		
Bersinger [30]	Low level [0.63 MoM]		
Elshabacy [4]	Low PAPP-A combined with Doppler [1.1 µg/mL]		
Hoseini [32]	Low PAPP-A [0.75 MoM]		
Chandrasekaran [33]	Low PAPP-A in early PE [0.72 MoM]		
Saruhan [34]	Low predictive value [< or = 10th percentile]		
Kirkegaard [35]	Low predictive value [0.5 MoM]		
Masihi [39]	Low level [0.2 MoM]		
Nicolaides [42]	Low level [0.772 MoM]		
Sifakis [13]	Low level of placental expression		
Mesdaghi-Nia [51]		High placental thickness [4 cm]	
Alkafrawy [52]		High placental thickness in PE [>1.2 MoM]	
Vachon-Marceau [19]		High in PE/low in IUGR [>1.2 MoM in PE compared to 1.03 MoM in IUGR and normal pregnancies]	
Najafian [53]		High in PE [4 cm]	
Fatma Mortada Ali [54]		Significantly decreased placental thickness, including IUGR [6 mm in trim I]	
Kishwara [55]		Low placenta thickness in PE [1.51 cm]	
Karthikeyan [58]		Low placenta thickness in IUGR [23.78 mm]	
Sun [57]		High in PE [4 cm]	

PE—pre-eclampsia; IUGR—intrauterine growth restriction.

## Data Availability

All data are available from the corresponding author upon reasonable request.

## Abbreviations

The following abbreviations are used in this manuscript:

PE	pre-eclampsia
IUGR	intrauterine growth restriction
PAPP-A	pregnancy-associated plasma protein-A
sFlt-1	soluble fms-like tyrosine kinase-1
AUC	area under the curve
PlGF	placental growth factor
IGFBP	insulin-like growth factor-binding protein
DNA	deoxyribonucleic acid
ROC	receiver operating characteristic
BMI	body mass index
PI	pulsatility index
AFP	alpha fetoprotein
HELLP	hemolysis, elevated liver enzymes, and low platelets
cfDNA	cell-free DNA
MVs	microvesicles
